# Fellow-Workers and the Roll of Honour

**Published:** 1915-06-26

**Authors:** 


### ROUND THE WORLD.

[The Editor invites Early Information and Contributions Marked "Fellow-Workers."]

### Fellow-Workers and the Roll of Honour.

Lieutenant-Colonel John William Jessop, whose death abroad has lately been reported, was the commanding officer of the 4th Lincolnshires (T.F.), and in civil life had been for some years in practice as a medical man at Horncastle. Leaving his home in Devonshire to study medicine he entered as a student at St. Bartholomew's Hospital, and qualified L.R.C.P., M.R.C.S., in 1889. Settling in Horncastle the late officer became surgeon to the local dispensary, Poor-Law medical officer for the district, and medical officer to the Post Office. A keen Volunteer, he took a commission in the Horncastle Company before the days of the Territorials, and, continuing his work after the reorganisation of the Auxiliary Forces, was given command of his battalion three years ago.

Another keen combatant officer who left medical work to serve at the Front was the late Second-Lieutenant J. E. Bruce Miller, whose death from wounds occurred recently. The outbreak of war found Mr. Bruce Miller a student of Edinburgh University, where he belonged to the Artillery unit of the O.T.C. The prospect of early employment abroad induced him to accept a commission in the King's Royal Irish Rifles (5th Battalion).

The Welsh Metropolitan War Hospital is being administered in the buildings of the Cardiff Mental Hospital, under the command of the heretofore medical superintendent Dr. E. Goodall, who has been given the rank of lieutenant-colonel. The commandant retains his former staff on a military basis, and Drs. E. B. C. White, H. D. Harrison, and C. H. G. Ramsbottom will rank as majors whilst working at this institution, which will accommodate some 900 wounded.

Lieutenant-Colonel Edwin Goodall, R.A.M.C., is one of our best known alienists, and as medical superintendent of the Cardiff Mental Hospital has given keen attention to various important problems, pathological, clinical, and administrative, of asylum work. A student of Guy's Hospital, he qualified in 1885, became an M.D. Lond. three years later, and was elected to the Fellowship of the Royal College of Physicians in 1903. Before being appointed to the responsible post in which he has found such distinction Lieutenant-Colonel Goodall had much experience of insanity as resident clinical assistant at Bethlem Royal Hospital, assistant medical officer at the West Riding Asylum, Wakefield, and medical superintendent to the Joint Counties Asylum at Carmarthen.

Major Edward Barton Cartwright White, R.A.M.C., has been the senior assistant medical officer at the Cardiff Mental Hospital. Qualifying L.R.C.P., M.R.C.S., from the London Hospital in 1908, he was for a time house physician at Bethlem Royal Hospital. Major White has investigated the relation between pituitary and suprarenal growths and insanity.

Lieutenant-Colonel William Aldren Turner, R.A.M.C. (T.), who has lately been promoted to his present rank, has been doing excellent work in the investigation of cases of nervous disorder arising from the conditions of warfare to-day. Recently he published an account of his observations on shell-shock, traumatic neuroses, and various forms of mental disturbance incidental to modern war. Lieutenant-Colonel Turner, who has for many years occupied a place in the front rank of London neurologists, is physician to King's College Hospital and to the National Hospital for Paralysis and Epilepsy in Queen Square.

Many Guy's men will have noted with affectionate approval the promotion of their old teacher, Mr. Charters James Symonds, to the rank of Lieutenant-Colonel in the R.A.M.C. (T.). Now consulting surgeon to Guy's Hospital, with which he maintained an active connection for many years, Lieutenant-Colonel Charters Symonds had a brilliant career as a student, winning the gold medal at the B.S. Lond. (1877), and the gold medals in forensic medicine and obstetric medicine with first-class honours in medicine at the M.B. Lond. (1877).

Lieutenant David Galloway Watson, R.A.M.C., reported died of wounds, was stricken down whilst leading a stretcher party on the Continent, where he had been working for some time. An Edinburgh University man, he qualified M.B., Ch.B. in 1913, after a student's career in which he had taken a prominent part in university matters. After qualification he obtained a provincial hospital appointment, but joined the R.A.M.C. with a temporary commission a few days after the declaration of war last summer.

It is stated that Surgeon Thomas Louis Grenet Stewart, R.N., who was fatally wounded recently, is the first naval surgeon to have been killed in actual fighting, although unfortunately a number have lost their lives in other ways. The late Mr. Stewart qualified at Glasgow by taking the M.B., Ch.B. degrees there in 1911. Subsequently he held resident posts at the Borough Hospital, Birkenhead, and the Brownlow Hill Infirmary, Liverpool. When the war broke out he obtained a temporary commission in the Royal Navy.

Mr. Charles Eric Sweeting, who, as announced in The Hospital (June 19, 1915), has been appointed Médecin-Chef to the Hôpital Militaire Anglais at Limoges, qualified L.R.C.P., M.R.C.S. in 1911, and subsequently took the degrees of M.B., B.S. Lond., and the Fellowship of the Royal College of Surgeons. Before being elected surgeon to the West Norfolk and Lynn Hospital he held resident appointments at St. Mary's Hospital, and Queen Mary's Hospital for Children at Carshalton.

Mr. John Percy Lockhart-Mummery, the well-known authority on rectal troubles, is now special surgeon for diseases of the bowel to the Fulham and Homerton Military Hospitals. A student of Cambridge and St. George's, he qualified in 1899 and became an F.R.C.S. Eng. in the following year. His present appointments include those of senior surgeon to St. Mark's Hospital and surgeon to the Queen's Hospital for Children at Bethnal Green. Mr. Lockhart-Mummery was formerly demonstrator of operative surgery and house surgeon at St. George's Hospital.

